# Arsenic and Sesquicarbonate of Ammonia in Ague

**Published:** 1862-06

**Authors:** Edward Adamson

**Affiliations:** M. D. Edin.


### ARSENIC AND SESQUICARBONATE OF AMMONIA IN AGUE. By Edward Adamson, M.D. Edin.

As a substitute for the preparations of cinchona in the treatment of ague, I doubt if there be any remedy more efficacious and trustworthy than the combined use of sesquicarbonate of ammonia and liquor arsenicalis. In ten cases, all adult males, treated solely with this remedy, it proved uniformly successful. Thus: in one case only—a quotidian—was its administration followed by two paroxysms; in seven other cases (one quotidian and six quartan) only one paroxysm subsequently recurred; and not one in the remaining two cases (one tertian and one quartan). As yet I have had opportunity of using these combined drugs in only two other cases of adult males; but, as both these cases had long resisted quinine, though readily yielding to the ammonia and arsenic, I do not include them in the above list. The quantity of the sesquicarbonate usually given was five grains, dissolved in one ounce of water, with the addition of five minims of liquor arsenicalis; this dose being repeated *every two* or *every three hours,* according to the frequency of the paroxysms. In no case did any inconvenience result, save some degree of griping in one patient, in whom, as well as in three others, there was slight itching about the eyelids, and this was not spontaneously complained of but in two cases. Doubtless the real antiperiodic power is to be ascribed to the arsenic, which the sesquicarbonate, while exaggerating perhaps its efficacy, renders more easily tolerated by the system.—*Edinburgh Medical Journal.*

